# Molecular and Functional Imaging for Detection of Lymph Node Metastases in Prostate Cancer

**DOI:** 10.3390/ijms140713842

**Published:** 2013-07-03

**Authors:** Ansje Fortuin, Maarten de Rooij, Patrik Zamecnik, Uwe Haberkorn, Jelle Barentsz

**Affiliations:** 1 Department of Radiology, Nijmegen Medical Center, Radboud University, P.O. Box 9101, 6500 HB Nijmegen, The Netherlands; E-Mails: m.derooij@rad.umcn.nl (M.R.); p.zamecnik@rad.umcn.nl (P.Z.); j.barentsz@rad.umcn.nl (J.B.); 2 Department of Operation Rooms, Nijmegen Medical Center, Radboud University, P.O. Box 9101, 6500 HB Nijmegen, The Netherlands; 3 Department of Nuclear Medicine, University of Heidelberg, Im Neuenheimer Feld 400, Heidelberg 69120, Germany; E-Mail: uwe.haberkorn@med.uni-heidelberg.de

**Keywords:** molecular imaging, lymph node metastasis, prostate cancer, choline PET/CT, USPIO, PSMA, sentinel node imaging, DWI MRI, IMRT, SPECT

## Abstract

Knowledge on lymph node metastases is crucial for the prognosis and treatment of prostate cancer patients. Conventional anatomic imaging often fails to differentiate benign from metastatic lymph nodes. Pelvic lymph node dissection is an invasive technique and underestimates the extent of lymph node metastases. Therefore, there is a need for more accurate non-invasive diagnostic techniques. Molecular and functional imaging has been subject of research for the last decades, in this respect. Therefore, in this article the value of imaging techniques to detect lymph node metastases is reviewed. These techniques include scintigraphy, sentinel node imaging, positron emission tomography/computed tomography (PET/CT), diffusion weighted magnetic resonance imaging (DWI MRI) and magnetic resonance lymphography (MRL). Knowledge on pathway and size of lymph node metastases has increased with molecular and functional imaging. Furthermore, improved detection and localization of lymph node metastases will enable (focal) treatment of the positive nodes only.

## 1. Introduction

There is increasing knowledge of lymph node metastases in patients with prostate cancer. This knowledge helps to predict prognosis [1,2] and is important to optimize treatment planning [3].

### 1.1. Conventional Anatomic Imaging

The normal upper limit of the short-axis diameter of pelvic lymph nodes is 7–10 mm depending on the location. However, up to 80% of metastatic lymph nodes in prostate cancer have a short axis diameter smaller than 7 mm [3,4]. Therefore, it is not surprising that a limited sensitivity is reported for conventional computed tomography (CT) and magnetic resonance imaging (MRI), as they rely on the size criteria to differentiate between benign and malignant lymph nodes. A pooled sensitivity of 42% and 39% and pooled specificity of 82% and 82% for CT and MRI have been reported in a meta-analysis of Hovels *et al*. [5]. The used cutoff value varied between 5 and 15 mm. Hilton *et al.* showed that the sensitivity improved from 37% to 93% when lowering the threshold diameter from ≥10 mm to ≥4 mm, but this is at the cost of specificity which drops from 100% to 58% [4].

### 1.2. Pelvic Lymph Node Dissection (PLND)

PLND is widely used to access lymph node metastasis in prostate cancer. It can be omitted in patients with less than 5% risk of lymph node involvement, but is otherwise advised [5]. This technique is invasive and underestimates lymph node involvement, as in approximately 40%–50% of patients metastatic lymph nodes are found outside the standard resection area [6,7]. At the cost of higher complication rates, extended PLND shows better results. However, recently Joniau *et al.* have shown that a rather high number (13%) of metastatic lymph nodes are missed with extended PLND as well [8].

### 1.3. Rationale for Molecular and Functional Imaging

Because of the limitations of conventional anatomic imaging and PLND to assess lymph node metastases accurately, alternative strategies are needed. Research focuses on the ability of molecular and functional imaging to differentiate metastatic from benign lymph nodes *in vivo*, using direct or indirect methods. Direct methods assess metabolic behavior, expression of structures located at the cell membrane such as antibodies and receptors and molecular diffusion of metastases. Indirect methods trace the lymphatic route from the primary tumor or determine nano-particle incorporation in macrophages in non-metastatic lymph nodes.

In this article, we present a literature overview of the molecular and functional imaging techniques. See Table S1 for an overview of the imaging modalities and in short their pro’s and con’s.

## 2. Molecular and Functional Imaging

### 2.1. Planar Scintigraphy, Single-Photon Emission Computed Tomography (SPECT) and Gamma Probe

In scintigraphy and SPECT, a gamma ray emitting radionuclide coupled to a molecule that ensures specific accumulation to a tissue of interest (tracer) is admitted to the patient. These gamma rays are detected by an external gamma camera. With scintigraphy, two-dimensional images (2D) are reconstructed. With SPECT, the location of the tracer is calculated and a three-dimensional image (3D) is reconstructed. SPECT can be combined with conventional CT or MRI to add anatomical to functional information (SPECT/CT or SPECT/MRI).

A gamma probe is the simplest gamma ray detector. It is a device with a Geiger-Müller tube or scintillation counter. After injection of a radionuclide, in or near the tumor in the case of sentinel node imaging, the radioactivity can be located during surgery.

#### 2.1.1. Sentinel Lymph Node Imaging

With sentinel lymph node imaging a radiotracer is injected into the prostate under trans rectal ultrasound guidance (TRUS). Subsequently, the radiotracer enters the lymphatics. The first lymph node stations that are reached are called sentinel lymph nodes. These nodes can be detected prior surgery on planar scintigraphy or SPECT. During surgery the lymph nodes with radiotracer uptake can be detected with a gamma detecting probe [9].

The most commonly used radiotracer is ^99m^TC-nanocolloid. Nanocolloid accumulates in the sentinel lymph node [10], which can then be removed and analyzed by immunohistochemistry in more detail as compared to the standard pathological procedure. The rationale behind this procedure is that metastases reach these sentinel nodes first, before spreading further through the body, either lymphatic or hematogeneous. If the sentinel lymph node is free of tumor cells a further PLND can be avoided.

This technique has changed our knowledge on lymph node drainage of prostate cancer. New maps of lymph node drainage were conducted [11,12]. This showed 66% of patients had sentinel lymph nodes outside the used standard target volume for whole pelvic radiotherapy [12]. This may explain the variable and somewhat disappointing radiation results with radiation therapy [13].

In 2011, Sadeghi *et al.* performed a meta-analysis on sentinel node mapping in prostate cancer. The pooled detection rate was 94% [14]. The Augsberg group published the results of until now the largest study on sentinel node mapping [15]. Their reported detection rate was 98%. However, this reflects only the intraoperative detection of at least one sentinel node. In 24% of the patients not all the preoperatively visible sentinel nodes could be harvested during surgery. Also, in 40% of patients additional sentinel nodes were found intra operatively. Furthermore, when lymph nodes are fully replaced by metastasis, the afferent lymph will be directed to other nodes [16]. These nodes will not be positive on sentinel node imaging resulting in false-negative findings. A sub analysis of the Augsberg group showed a mean false-negative rate of 6%. The false negative rate was shown to correlate with the Gleason Score: In patients with a Gleason Score of 4 + 3 the false negative rate was 4%, whereas the false negative rate in patients with a Gleason Score of >8 was 14% [15]. Therefore, sentinel lymph node imaging seems to be of value mainly in primary prostate cancer patients with a Gleason Score of 8 or lower.

#### 2.1.2. Radiolabeled Monoclonal Antibodies and Small Molecules

Many different antibodies have been under research for therapy and imaging of prostate cancer and its metastases [17]. The best-known target is a cell surface protein named prostate specific membrane antigen (PSMA). PSMA is extremely over expressed in prostate cancer and its metastases compared to other tissues [18]. Only one radiolabeled monoclonal antibody is FDA approved, 111-indium capromab pendetide (Prostascint), imaged on planar scintigraphy or SPECT/CT. Although initial results showed improvement over conventional imaging, sensitivity and specificity are limited [17,19–21]. Recently, Hardie *et al.* combined SPECT/CT with diffusion weighted MRI and found an increased sensitivity and specificity by the addition of MRI [22]. Nevertheless, the main disadvantage of Prostascint is the fact that this agent targets PSMAs intracellular domain. Therefore, imaging becomes only accessible upon apoptosis or necrosis and not in viable tissue [17,23]. PSMA presents also a large extracellular target and therefore, main research is now focused on the extracellular domain [24]. Recently, a new antibody J591 directed against the extracellular domain of PSMA and labelled with ^89^Zr showed promising results in tumor bearing animals [25]. In general, antibodies have a low plasma clearance associated with a long circulation time and therefore, a high background activity leads to useful contrast only in late images. Furthermore, the relative high molecular weight limits tissue penetration, especially in tumors with a high interstitial pressure. Therefore, besides antibodies, small molecules have been applied as well. Since PSMA shows enzyme activity, many small molecule inhibitors have been developed, which have been used for the development of tracers for diagnosis and therapy. A first-in men study with high affinity PSMA-avid small molecules, ^123^I-MIP-1072 and ^123^I-MIP-1095, was recently published by Barrett *et al* showing promising results for detection of lesions with excellent pharmacokinetic and pharmacodynamics profiles [26]. Currently, ^99m^Tc-MIP-1404, another PSMA small molecule ligand, is under evaluation in an international Phase 2 study in men scheduled for radical prostatectomy at high risk for lymph node involvement. The study will evaluate the ability of MIP-1404 to detect disease, using histopathology as the gold standard (ClinicalTrials.gov Identifier: NCT01667536).

In summary, agents targeting the intracellular domain and which are sufficient for use in clinical practice seem limited. However, first studies with labeled small molecules targeting PSMA show promising initial results for diagnosis and treatment.

### 2.2. Positron Emission Tomography (PET)

In PET imaging, a positron emitting radionuclide (tracer) is admitted to the patient. Such a tracer emits indirectly a pair of gamma rays. In general, the tracer is combined to a compound used in body metabolism, such as glucose. The system detects the gamma rays and reconstructs 3D images of the radiotracer uptake in the body. Nowadays PET is often combined with CT to combine molecular and anatomical information. More recent PET can also be combined with MRI.

#### 2.2.1. FDG

Malignant tumors show an increased glycolytic activity, which is associated with changes in the expression of glycolysis-associated genes occurring during malignant transformation. The most important genes are those for glucose transporter subtype-1 and hexokinase. These changes in transport and phosphorylation are exploited for tumor imaging with ^18^F-fluorodeoxyglucose (FDG) by positron emission tomography (PET). FDG for PET studies of glucose metabolism was introduced as a consequence of autoradiographic and biochemical studies with glucose analogs in different tissues [27]. Therefore, in oncology the most common metabolic radiotracer is ^18^F-fluoro-2-deoxy-D-glucose (FDG), an analog of glucose. Unfortunately, in prostate cancer the results with FDG are disappointing [28–30], possibly because prostate carcinoma is a slow-growing tumor where glucose metabolism is not at all or only moderately increased, resulting in a low, not differentiating, uptake of FDG [28]. A further disadvantage for using FDG in prostate cancer is the renal excretion, which can obscure pelvic lymph nodes by accumulation of FDG in the bladder [30]. Therefore, FDG PET plays a limited role in the detection of lymph node metastases in prostate cancer.

#### 2.2.2. Choline

In prostate cancer most PET investigations are performed with choline derivates. Choline tracers are incorporated into tumor cells after transport and phosphorylation by choline kinase [31] that is up regulated in prostate cancer and prostate cancer metastases [32]. Choline analogs are transported into the cells. Subsequently, they are rapidly metabolized to phosphocholine or oxidized by choline dehydrogenase and betaine-aldehyde dehydrogenase to betaine. The latter occurs mainly in the liver and the kidneys. The phosphorylation of choline by choline kinase represents the first and obligatory step for the incorporation of choline into phosphatidylcholine. The resulting first metabolite, phosphocholine, is negatively charged and therefore, trapped within the cell [27].

Different tracers have been combined with choline. ^11^C-choline has the extra advantage that it shows only minimal urinary excretion and therefore, minimal activity in the bladder. The reported performance of PET/CT for LN metastasis detection varies from 19% to 80% for sensitivity and 82%–98% for specificity on a patient-by-patient analysis [33]. Results are good in larger nodes but sensitivity is limited in lymph nodes smaller than 7 mm [34]. Limited resolution affects the results and therefore also the utility. A practical disadvantage is the short half-life of ^11^C of 20 min and therefore requiring an onsite cyclotron.

**Figure 1:**
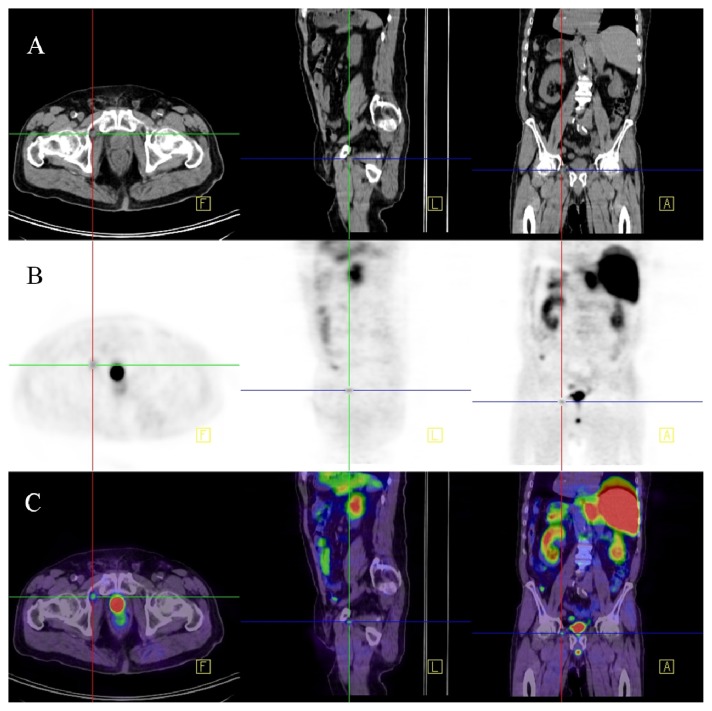
^18^F-choline PET-CT. Patient after radical prostatectomy with slowly increasing PSA (current PSA: 1.5 μg/L). The figure shows series of native CT images (**A**); PET images 60 min after the administration of ^18^F-choline (**B**) and fused PET-CT images (**C**) in transversal, sagittal and coronal views. The PET images show a small hot spot in the right inguinal region with increased isotope uptake. In the PET-CT fused images this spot can be identified as a small right inguinal lymph node highly suspect for a metastasis. (F: Transverse plane; L: Saggital plane; A: Coronal plane).

^18^F-choline has a half-life of the isotope of 110 min and is, as a consequence, more easily available. On the other hand, ^18^F-choline has greater urinary excretion than ^11^C-choline. Nonetheless, as pathological uptake begins one-minute post injection (before urinary excretion). This is less a problem than in FDG PET/CT. In benign prostate tissues, a decrease of tracer accumulation was observed with time, also leading to a higher contrast between tumour and normal tissue. It has been suggested that this is due to a dephosphorylation of ^18^F-phosphorylfluoro-choline by prostatic acid phosphatase, an enzyme that is specific for prostate tissues [35,36]. Normal prostate tissue, as well as prostate hyperplasia, contains higher levels of prostatic acid phosphatase than prostate carcinoma, which accepts phosphocholine and possibly also ^18^F-phosphorylfluoro-choline as a substrate. Theoretically ^18^F-choline has a somewhat higher resolution because of a shorter positron length path although the results are varying. Reported sensitivity and specificity are 10%–100% and 80%–100%, respectively, on a patient-by-patient analysis in primary disease [33]. Tilka *et al.* recently performed a lesion-based analysis in 1149 lymph nodes in 56 patients yielding a sensitivity of 40% and a specificity of 96% [37]. In recurrent disease the detection rate of metastases with choline PET/CT shows to be higher when PSA levels increase [37,38]. See Figure 1 for an example of ^18^F-choline PET-CT imaging.

In a recent study, simultaneous ^18^F-choline PET and MRI imaging in the prostate showed to be feasible [39] and can potentially improve the current results of choline PET imaging. Further research is warranted to see if this combination may also be feasible for lymph node imaging. Choline PET-CT is currently mainly helpful in the case of positive lymph nodes. However, a negative result has to be handled with care as sensitivity for small lymph node metastases has shown to be limited.

#### 2.2.3. Acetate

Acetate has, similar to choline, a role in fatty acid metabolism by participation in cytoplasmic lipid synthesis. Fatty acid synthase is an enzymatic protein that catalyses fatty acid biosynthesis and is overexpressed in prostate carcinoma compared to normal tissues [40]. The urinary excretion is minimal. Different tracers can be combined with acetate, but most research is performed with ^11^C-Acetate.

Haseebuddin *et al.* recently conducted a study in 107 patients with intermediate- or high-risk localized primary prostate cancer and negative conventional imaging findings for lymph node involvement [41]. They reported a sensitivity and specificity of 68% and 78%, respectively, on a per-patient basis using ^11^C-Acetate in patients with primary disease. Just as ^11^C-choline it has the practical disadvantage that the half-life of ^11^C is only 20 minutes, requiring an onsite cyclotron. Also a shown positive correlation between PSA and ^11^C-Acetate uptake limits clinical utility [42].

#### 2.2.4. ^68^Ga-Labelled PSMA Ligand

The latest development in PET imaging is ^68^Ga-labelled PSMA ligand Glu-NH-CO-NH-Lys(Ahx)-HBED-CC. This ligand targets PSMA, which is until now mainly a target in scintigraphy.

**Figure 2:**
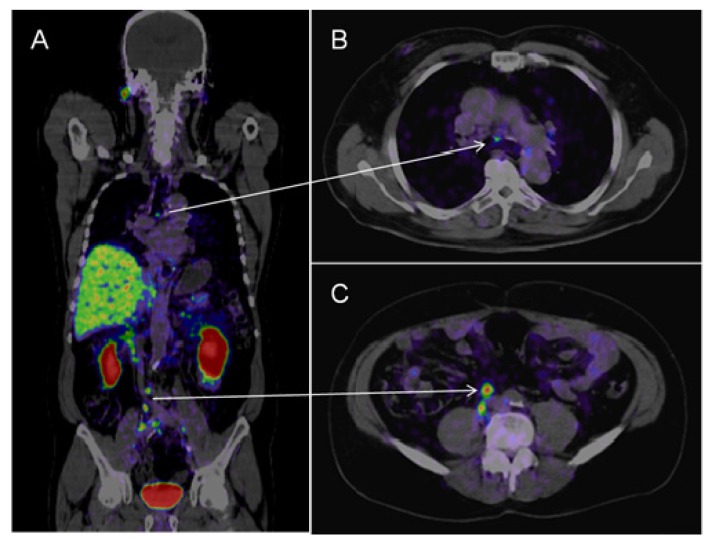

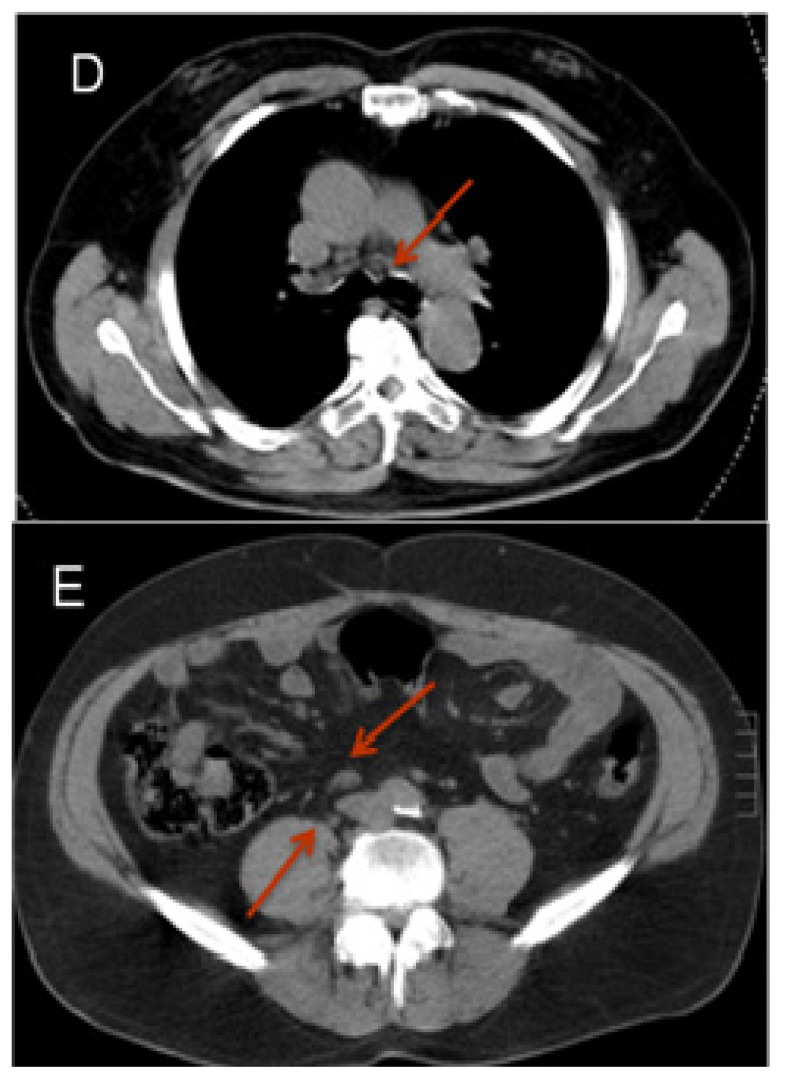
*^68^*Ga-PSMA-PET-CT. Patient with prostate cancer (status after brachy-therapy and bilateral iliac lymph node dissection, current PSA 21 μg/L). PET images were acquired after the administration of *^68^*GA-PSMA-Ligand (60 min thereafter). The figure shows fused images (PET-CT): On the coronal view (**A**) a pathologic isotope uptake in multiple lymph nodes in the right para-iliac region and infra-carinal (mediastinum) is clearly visible; the corresponding transversal images show the para-iliac (**B**) and infra-carinal (**C**) lymph nodes with elevated uptake of the tracer. The corresponding transversal native CT images present these suspect structures (**D**,**E**) as normal sized lymph nodes (marked by red arrows).

Afshar-Oromich *et al.* showed promising results in an initial study in 37 patients. They observed excellent contrast uptake, also in small lymph nodes (see Figure 2) [43]. In direct comparison to 18F labeled Choline, first results suggest that PSMA-targeted imaging performs better in detection of small lymph nodes in patients with low PSA values [44]. If these results are reproducible this may have significant clinical impact on diagnosing prostatic lymph node metastases.

### 2.3. Magnetic Resonance Imaging (MRI)

MRI is an excellent imaging modality for anatomical information, especially because it provides good contrast between different soft tissues. For this paper especially, the functional and molecular opportunities of MRI are of interest.

#### 2.3.1. Diffusion Weighted Imaging (DWI)

In DWI, molecular diffusion of water molecules is imaged (Brownian motion). The calculated apparent diffusion coefficient (ADC) map shows a quantification of the diffusion coefficient. Diffusion of benign and metastatic lymph nodes may differ from each other, providing a potential tool for differentiation. Few studies have been performed to assess its value for detection of lymph node metastases in prostate cancer. Eiber *et al.* analyzed lymph nodes of 29 prostate cancer patients with a short axis of >6 mm. Ten patients underwent lymphadenectomy with histological correlation of lymph nodes. The other 19 were followed up for a mean of 9 months (range 6–12 months). Malignant lymph nodes showed restricted diffusion resulting in a lower ADC-value compared to benign lymph nodes. Sensitivity and specificity were 86% and 85%, respectively, compared to a size-based analysis at a cutoff of 8 mm with sensitivity and specificity of 82% and 54%, respectively [45]. In 2011, this group showed similar results for DWI imaging in a smaller group of patients [46]. On the contrary, Budiharto *et al.* reported disappointing results in 2011 in a group of 36 high-risk prostate cancer patients with no shown lymph node metastasis on conventional imaging. Minimal axial diameter of included lymph nodes was 4 mm. All patients underwent an extended pelvic lymph node dissection. Sensitivity for lymph node region-based analysis was only 19% (specificity 98%). In a per-patient analysis the sensitivity increased to 43% (specificity 82%) [47]. Similar to Choline PET-CT the sensitivity of DWI is especially limited in small lymph nodes. Furthermore, Choline PET-CT seems to perform better in terms of specificity.

#### 2.3.2. Magnetic Resonance Lymphography (MRL)

**Figure 3:**
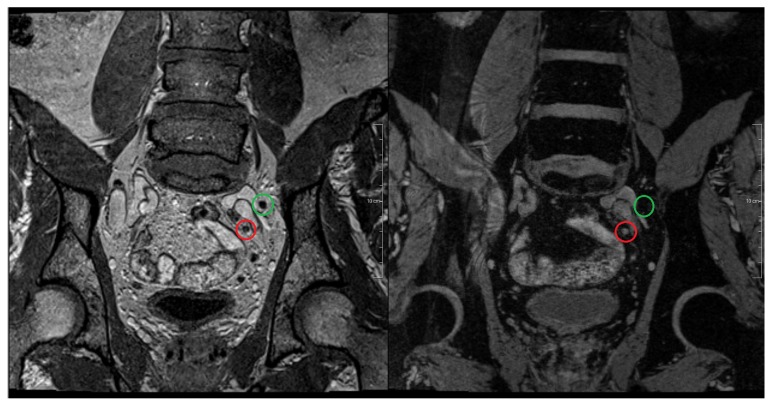
MR-lymphography at 3 Tesla using USPIO. Patient with prostate cancer and lymphatic metastases. Left image shows the morphological T1 weighted coronal sequence with two small lymph nodes near to the left common iliac artery. Right image shows the corresponding T2* weighted coronal image 24 h after the intravenous administration of an USPIO agent (ferumoxtran-10). The lymph node marked by the green circle shows an USPIO uptake and therefore a signal loss in the T2* image suggesting normal lymphatic tissue. The lymph node marked by the red circle does not show any USPIO uptake and therefore shows a bright signal on the T2* image. This lymph node is highly suspect for metastasis.

In MRL, normal lymph node tissue can be distinguished from metastatic tissue by labeling macrophages in normal lymph node tissue using ultra small super paramagnetic iron oxide (USPIO) particles. Benign lymph nodes will therefore undergo a change in their magnetic properties. On T2* (iron sensitive) images, benign lymph nodes lose their signal in contrast to metastatic lymph nodes who have a bright signal (Figure 3). In this way, even small metastases in normal sized lymph nodes can be detected [34,48]. Studies, with respectively 80, 50 and 375 patients with primary prostate cancer included, have reported a sensitivity and specificity of 88%–92% and 93%–98%, respectively, for MRL with ferumoxtran-10 as USPIO agent [48–50]. Triantafyllou *et al.* [51] recently published a study including 75 patients with bladder and/or prostate cancer. In this study only patients with normal sized lymph nodes were included and a meticulous histopathological analysis of each lymph node was applied. This resulted in a more limited sensitivity and specificity (55%–58% and 83%–85%, respectively). The reported inter observer variability was furthermore not negligible, possibly also skewing the results [52]. In previous work the same group added a DWI sequence to MRL for faster detection of lymph nodes. This significantly shortened study analysis without compromising the study results [53]. MRL further improved knowledge about the lymph node pattern of spread. Heesakkers *et al.* showed that in 41% of the patients, histologically confirmed lymph nodes were located only outside the area of standard lymph node dissection. In another 41% of patients, histologically confirmed metastatic lymph nodes were found both inside and outside this area [6]. Meijer *et al.* showed that 53% of primary prostate cancer patients, with intermediate and high risk with non-enlarged lymph nodes, had at least one MRL positive lymph node outside the clinical target volume for elective pelvic irradiation as defined by the radiation therapy oncology group [54]. In patients with biochemical recurrence, after radical prostatectomy this percentage increased to 79% [55]. Furthermore, thanks to MRL, the knowledge about the size of lymph node metastasis is improved [34,48,51]. Although the results with MRL are promising, USPIO agents, like ferumoxtran-10, are currently not available for clinical routine use.

## 3. Focal Treatment Options for Lymph Node Metastases

Improved detection and localization of lymph node metastases opens the door for new treatment options.

There is evidence that patients can benefit from lymph node dissection in a therapeutic way [56–59]. Sentinel lymph node imaging and MRL improved our knowledge on where to expect metastatic lymph nodes, which can improve the results of a dissection. Limited or isolated lymph node metastases detected with molecular and functional imaging techniques may be treated with focal therapy. First reports on 11C-choline guided surgery show encouraging results [60,61], as is the case for 11C-choline guide robotic image-guided stereotactic radiotherapy [62].

Also, studies with MRL guided intensity-modulated radiotherapy have shown to be feasible and to eliminate metastatic lymph nodes [63,64]. As the results with ^68^Ga-labelled PSMA ligand PET-CT are promising, also in small lymph nodes, ^68^Ga-labelled PSMA ligand PET-CT guided focal therapy is expected.

## 4. Conclusions

Conventional CT and MRI play a limited role in lymph node detection in prostate cancer. Pelvic lymph node dissection therefore is a widely used method to evaluate lymph node metastases. This, however, is an invasive technique, which often underestimates the real extent of lymphatic metastases. In the last decade, extensive research has been performed in the field of molecular and functional imaging trying to solve this problem. The sentinel node imaging increased the knowledge about the lymphatic drainage pathway. However, the risk of false negative results using this technique is considerable.

The first generation of radiolabeled antibodies used for lymph node imaging showed limited sensitivity and specificity, but the second and third generations of tracers are available now, and the preliminary results are promising.

Choline PET/CT is already an established technique for lymph node imaging in patients with prostate cancer, although sensitivity is limited in normal sized lymph nodes. First results on 11C-choline guided therapy are promising.

Excellent results have been shown using USPIO enhanced MRL. This technique had been leading to further improvement of the knowledge on the pathway of spread and size of metastatic nodes. For clinical practice this is a promising technique, as well are the results with MRL guided focal therapy promising. Unfortunately, USPIO agents are clinically not available at the moment.

## Supplementary Information
