# Supplementary material for: Molecular and Functional Imaging for Detection of Lymph Node Metastases in Prostate Cancer
Source: Int J Mol Sci. 2013 Jul 3;14(7):13842–57. doi: 10.3390/ijms140713842 (PMC3742221; doi:10.3390/ijms140713842)
Supplement: Supplementary file 1 [file ijms-14-13842-s001.pdf]

Supplementary Information

Table S1. Pro's and Con's of imaging techniques in detecting prostate lymph node metastases.

Modality	Technique	Pro	Con
**Conventional imaging**			
CT	Anatomical imaging using CT	Fast imaging<br>Low costs	Rely on size criteria only Radiation risk
MRI	Anatomical imaging using MRI	No radiation risk	Rely on size criteria only
**Planar scintigrapy, SPECT and gamma probe**			
Sentinel lymph node imaging with scintigraphy or SPECT	Gamma ray emitting radionuclide coupled to tracer (e.g. using ^{99m}TC-nanocolloid), detected by external gamma camera to form 2D (scintigraphy) or 3D (SPECT) images	Provides anatomical and functional information when combined with CT/MRI.<br>Valuable for Gleason 8 or lower	During surgery additional sentinel nodes in 40%<br>Full metastatic lymph nodes can become invisible resulting in false negatives
Radiolabeled monoclonal antibodies	Radiolabeled monoclonal antibodies targeted to PSMA (e.g. 111-indium capromab penetide, ^{89}Zr-J591). Radiolabeled small molecules	Possibility to target prostate cancer and its metastases specifically<br>Possibilities for targeting treatment<br>Excellent pharmacokinetic and pharmacodynamics profile	Only 1 FDA approved radiolabeled monoclonal antibody (Prostascint).<br>Only accessible in case of necrosis and apoptosis (first generation).<br>Only very early results, clinical value has to be established

Table S1. *Cont.*

Modality	Technique	Pro	Con
**Positron emission tomography (PET)**			
FDG	Detects increased glucose metabolism in most tumors	Good results in other tumors	Low uptake of glucose in prostate cancer.<br>Renal excretion can obscure pelvic lymph nodes.
^{11}C-Choline	Choline kinase upregulated in prostate cancer and metastases	Minimal urinary excretion	Low sensitivity small lymph nodes.<br>Short half-life (20 min) requires onsite cyclotron.
^{18}F-Choline	Choline kinase upregulated in prostate cancer and metastases	Longer half-life (110 min).<br>Possibility of PET/MRI in future to improve resolution	Greater urinary excretion than ^{11}C-Choline
^{11}C -Acetate	Acetate role in fatty acid metabolism. Fatty acid synthase overexpressed in prostate cancer	Minimal urinary excretion	Short half-life (20 min) requires onsite cyclotron.<br>^{11}C –Acetate uptake shows a positive correlation with PSA.
^{68}Ga-labelled PSMA ligand	Ligand targeted to PSMA	Good contrast uptake in small lymph nodes.<br>Promising results with lower PSA values.	Only early results available.
**Functional and molecular MRI**			
Diffusion weighted imaging (DWI)	Diffusion of water molecules (Brownian motion), diffusion coefficient (ADC) is calculated	Diffusion in metastatic lymph nodes differs potentially from benign lymph nodes	Results for smaller lymph nodes are disappointing.<br>Overlap in ADC value.
Magnetic resonance lymphography (MRL)	Uptake of ultra-small super paramagnetic iron oxide (USPIO) (e.g. ferumoxtran-10) by macrophages in normal lymph node tissue imaged with T2* images	Uptake in normal lymph nodes, therefore no minimal uptake in metastases required and also very small lymph node metastases can be detected.	Ferumoxtran-10 is currently not available for clinical routine use.
